# Direct but not indirect co-culture with osteogenically differentiated human bone marrow stromal cells increases RANKL/OPG ratio in human breast cancer cells generating bone metastases

**DOI:** 10.1186/1476-4598-13-238

**Published:** 2014-10-21

**Authors:** Chiara Arrigoni, Paola De Luca, Mara Gilardi, Sara Previdi, Massimo Broggini, Matteo Moretti

**Affiliations:** Cell and Tissue Engineering Lab, Gruppo Ospedaliero san Donato Foundation, Via R.Galeazzi 4, 20161 Milano, Italy; Cell and Tissue Engineering Lab, IRCCS Istituto Ortopedico Galeazzi, Via R.Galeazzi 4, 20161 Milano, Italy; Molecular Pharmacology Laboratory, IRCCS Istituto di ricerche farmacologiche Mario Negri, via La Masa 19, Milano, Italy

**Keywords:** Bone metastasis, *In vitro* co-cultures, Gene expression analyses, RANKL pathway, Heterotypic cell contacts

## Abstract

**Background:**

Bone metastases arise in nearly 70% of patients with advanced breast cancer, but the complex metastatic process has not been completely clarified yet. RANKL/RANK/OPG pathway modifications and the crosstalk between metastatic cells and bone have been indicated as potential drivers of the process. Interactions between tumor and bone cells have been studied *in vivo* and *in vitro*, but specific effects of the direct contact between human metastatic cells and human bone cells on RANKL/RANK/OPG pathway have not been investigated.

**Findings:**

We directly co-cultured bone metastatic human breast cancer cells (BOKL) with osteo-differentiated human mesenchymal cells (BMSCs) from 3 different donors. BMSCs and BOKL were then enzymatically separated and FACS sorted. We found a significant increase in the RANKL/OPG ratio as compared to control, which was not observed in BOKL cultured in medium conditioned by BMSCs, neither in BOKL directly cultured with fibroblasts or medium conditioned by fibroblasts. Direct co-culture with osteo-differentiated BMSCs caused BOKL aggregation while proliferation was not affected by co-culture. To more specifically associate RANKL expression to osteogenic differentiation degree of BMSCs, we determined their osteogenic markers expression and matrix calcification relative to osteoblasts and fibroblasts.

**Conclusions:**

In conclusion, our co-culture model allowed to demonstrate for the first time that direct contact but not paracrine interactions between human metastatic breast cancer cells and bone cells has a significant effect on RANKL/OPG expression in bone metastatic cells. Furthermore, only direct contact with the bone microenvironment induced BOKL clustering without however significantly influencing their proliferation and migration.

**Electronic supplementary material:**

The online version of this article (doi:10.1186/1476-4598-13-238) contains supplementary material, which is available to authorized users.

## Findings

### Introduction

Bone metastases arise in nearly 70% of patients with advanced breast cancer, indicating bone as a favorable microenvironment for metastases, which onset dramatically alters physiological bone turnover, leading to bone lysis. It has been reported [1] that this can be caused by the up-regulation of RANKL to OPG ratio, causing an increased osteoclastogenesis [2], although the mechanisms inducing RANKL/OPG modification are not clear [1, 3].

Recently, interactions between metastatic cells and metastatic niche cells have been indicated as potential drivers of the metastatic process [4]. Thus, to understand the cross-talk between metastatic breast cancer cells and bone microenvironment, many *in vitro* co-culture models have been established [5–10]. The majority of these [5, 6] involve human tumor cells cultured with mouse osteoblasts, but osteoblasts from different species have different characteristics [11] and mechanisms underlying metastasis formation can be diverse between mouse and human [12]. Moreover, while some studies reported the culture of human tumor cells in conditioned medium from human bone cells, highlighting only soluble factors effects [7, 8], only a few works [9, 10] implemented direct contact between human tumor and bone cells. However, gene expression analyses on individual cell populations have not always been performed [9].

Our work was thus aimed at investigating, by means of direct co-culture, the interactions between a line of fluorescently tagged bone metastatic human breast cancer cells, MDA-MB231-BO-KL (BOKL) and a bone microenvironment represented by osteo-differentiated primary human bone marrow stromal cells (BMSCs), in terms of modifications on RANKL/RANK/OPG expression, cell proliferation and migration.

#### Direct contact with bone-like cells induced RANKL/OPG up-regulation in metastatic cells

To investigate whether specific direct contact between bone and tumor cells is involved in RANKL/OPG ratio modifications, we compared gene expression of BOKL after 3 days of direct co-culture with osteo-differentiated BMSCs (co-culture BMSCs) with that of BOKL cultured in conditioned medium from osteo-differentiated BMSCs (CM BMSCs) and that of BOKL in direct or indirect co-culture with fibroblasts, non osteogenic cells (co-culture MRC-5, CM MRC-5). Based on literature data showing that cell populations with equivalent capacity to form bone metastasis also share equal expression of genes related to bone invasion and metastasis [13], we chose to use a single bone seeking clone [14], with equivalent bone metastatic potential as compared to other bone seeking clones [13]. BMSCs were harvested from 3 patients undergoing hip surgery, after informed consent, and were differentiated in osteogenic medium (OM), as described [15]. BOKL and BMSCs after direct co-culture with BMSCs or MRC-5 were retrieved by enzymatic digestion and separated by FACS sorting. To exclude modifications in gene expression caused by the separation procedure, gene expression levels were normalized respective to those of BOKL grown in GM and then subjected to the same separation procedure (control 1). CM for indirect co-culture was harvested from the same BMSCs and MRC-5 used in direct co-cultures and gene expression of BOKL in CM was normalized respective to BOKL grown in GM (control 2). All materials and methods are described in Additional file 1: Materials and methods.

**Figure 1:**
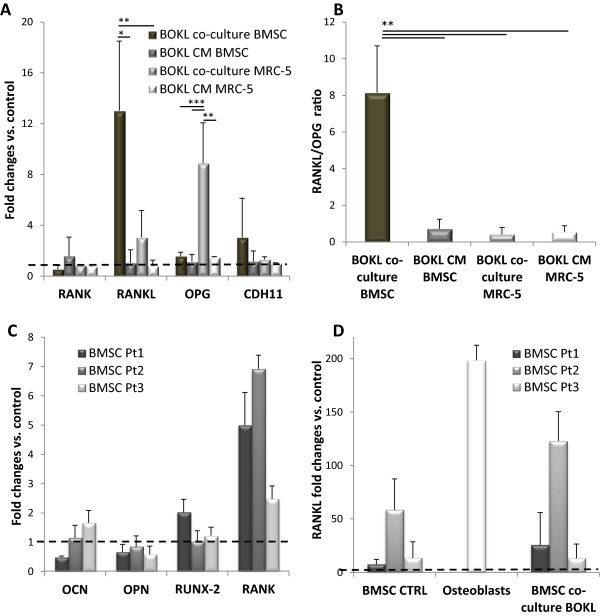
**Gene expression of BOKL and BMSCs from different patients cultured for 3 days in direct contact. A)** Expression of genes related to RANK/RANKL/OPG pathway and CDH11 in BOKL after direct co-culture with osteo-differentiated BMSCs (BOKL co-culture BMSC) compared with BOKL cultured in medium conditioned by the same osteo-differentiated BMSCs (BOKL CM BMSC), with BOKL after direct co-culture with fibroblasts (BOKL co-culture MRC-5) and with BOKL cultured in medium conditioned by the same fibroblasts (BOKL CM MRC-5). *: p < 0.05, **: p < 0.01 and ***: p < 0.001. Fold changes were calculated respective to control 1 or 2 (expression set to 1, dotted line). Control 1: BOKL grown in GM and subjected to enzymatic digestion and FACS sorting, used for BOKL from both direct co-cultures, control 2: BOKL grown in GM, used for BOKL in both CMs. **B)** RANKL/OPG ratio, for BOKL after direct co-culture with BMSCs and fibroblasts (BOKL co-culture BMSC and BOKL co-culture MRC-5) and in BOKL cultured in medium conditioned by BMSCs and fibroblasts (BOKL CM BMSC and BOKL CM MRC-5). **: p < 0.01. **C)** Gene expression of osteocalcin (OCN), osteopontin (OPN), RUNX-2 and RANK in BMSCs from three different patients (Pt1, Pt2, Pt3) after direct co-culture with BOKL. Fold changes were calculated respective to the same BMSCs before co-culture (expression value set to 1, dotted line). **D)** RANKL expression of BMSCs before (BMSC CTRL) and after direct co-culture (BMSCs co-culture BOKL), relative to three different patients, compared to that of osteoblasts. Fold changes were calculated respective to fibroblasts (expression set to 1, dotted line). All error bars represent standard deviations of at least three different experiments.

Results of PCR analyses showed a strong and statistically significant upregulation (13 fold, p < 0.05) of RANKL in BOKL co-culture BMSCs as compared to control 1 but not in BOKL CM BMSCs and BOKL CM MRC-5 (Figure 1A, p < 0.05 and p < 0.01, respectively). A small increase (not statistically significant) in RANKL was observed in BOKL co-culture MRC-5 where a significant upregulation of OPG was found (Figure 1A), differently from all other conditions. Altogether our results show that RANKL/OPG ratio (a more useful indicator of effectively available RANKL) in BOKL co-culture BMSCs is 7 fold increased (Figure 1B) relative to control, and significantly higher (p < 0.01) than all other conditions, where no increase was found, demonstrating that direct, specific contact between tumor and bone cells can modify RANKL/OPG ratio. This suggests that among other factors hypothesized in the literature [1], heterotypic interactions between metastatic and bone cells can be involved in RANKL/OPG imbalance.

We demonstrated a specific activation of RANKL/OPG and not a generalized change in gene expression, since CDH11, known to be highly expressed in bone-seeking clones [16], and RANKL receptor RANK were not significantly modified in BOKL after direct or indirect co-cultures (Figure 1A). We also analyzed gene expression of BMSCs after direct co-culture and we found that average value of RANKL and osteogenic differentiation markers expression did not significantly differ between osteo-differentiated BMSCs before and after direct co-culture. However, 1 out of 3 patients derived osteo-differentiated BMSCs (patient 2) showed a 4-fold RANKL up regulation after direct co-culture, due to the high biological variability of primary human cells (Figure 1C, D). RANK expression was instead significantly upregulated in BMSCs after co-culture with tumor cells (Figure 1C, p < 0.01 as compared to BMSCs before co-culture), differently from MRC-5, which showed no expression of RANK before or after co-culture (data not shown), confirming a specific activation of the RANKL/OPG/RANK pathway caused by the crosstalk between tumor cells and bone microenvironment.

The separation of metastatic cells from bone-like cells was based on fluorescent labeling of cells before co-culture, giving a particular reliability to our results. Another study analyzed gene expression of tumor cells in direct co-culture with BMSCs [10], separating the two cell populations on the basis of epithelial markers expression. It is however known that highly aggressive breast cancer cells in co-culture with BMSCs can undergo epithelial-to-mesenchymal transition [17], causing a loss of epithelial markers and consequently affecting cell separation reliability.

#### Direct co-culture effects on BOKL aggregation, migration and proliferation

**Figure 2:**
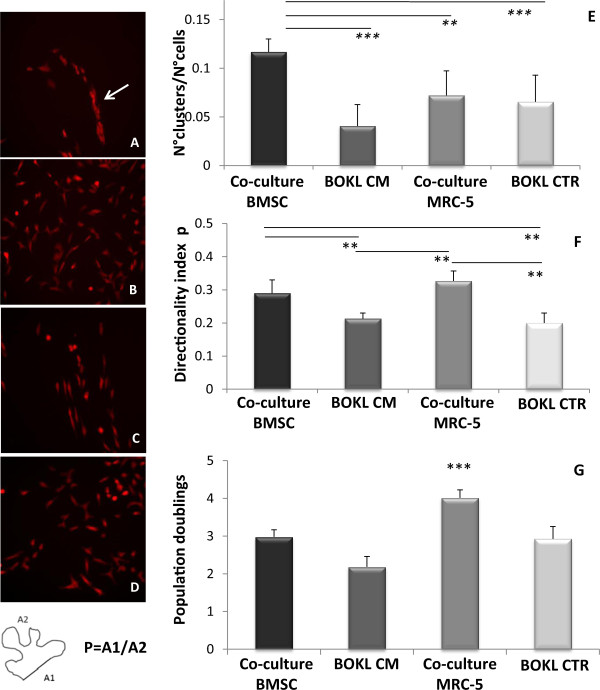
**Cell migration, aggregation and proliferation in different conditions.** Fluorescence images of: **A)** BOKL in direct co-culture with BMSCs (arrow indicates a cell cluster), **B)** BOKL cultured in CM BMSCs, **C)** BOKL in co-culture with MRC-5 and **D)** BOKL in GM. All the images have been taken after 3 days of culture. **E)** Quantification of the cell aggregation, expressed as the average number of clusters normalized per total cell number. Clusters were defined as groups of at least four cells in contact, as already reported for micrometastasis [20]. ***: p < 0.001, **p < 0.01 vs. other conditions. **F)** Quantification of the directionality of the cell movement, expressed as index P. P is defined as A1/A2, where A1 is the distance between the position of the cell in the last image and that in the first. A2 is the sum of distances travelled by the cell in all the images [21]. Values of P near 1 indicate a directional movement. **: p < 0.01 vs. other conditions. **G)** Proliferation of BOKL after 3 days of direct co-culture with BMSCs or MRC-5 and after 3 days in culture with conditioned medium from BMSCs (CM) or control medium (CTR), ***: p < 0.001. All error bars represent standard deviations of at least three different experiments.

With the aim to analyze effects of direct contact between bone-like cells and BOKL on aggregation, migration and proliferation, time lapse analyses were performed, revealing an evident BOKL aggregation in direct co-culture with osteo-differentiated BMSCs, not noticeable in the other conditions, as shown in fluorescence images (Figure 2A, B, C, D). This phenomenon resembled cell filing, already described in pathological tissues bearing metastases [5]. Interestingly, BOKL clustering did not occur in co-culture with MRC-5, suggesting that this can represent a specific effect of the direct contact with bone cells. Cluster number normalized to total cell number was indeed significantly higher in BOKL co-culture BMSCs, as compared to other conditions (p < 0.001, Figure 2E). We observed a preferential direction of cell migration in direct co-culture as compared to culture with CM or GM (p < 0.01, Figure 2F), probably due to cell contact guidance [18] and not to specific characteristics of cells that composed the monolayer, being this effect evident also in co-culture with MRC-5. Figure 2G reports the quantification of BOKL proliferation in the different conditions, showing no significant differences between BOKL co-culture BMSCs and BOKL CM BMSCs. BOKL grown in 100% CM did not show a lower degree of proliferation as compared to control 2, indicating that CM can support BOKL growth. On the other hand, BOKL in contact with fibroblasts showed a significantly higher (p < 0.001) proliferation, accordingly to literature results showing how the release of specific factors (such as interleukin-6) by fibroblasts can increase the growth of breast cancer cells [19].

#### Characterization of bone-like microenvironment

**Figure 3:**
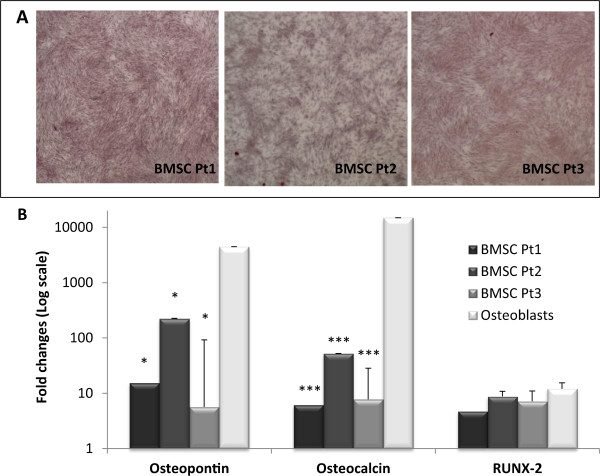
**Characterization of the bone-like microenvironment.** Characterization of the osteo-differentiation level of BMSCs from three different patients cultured for 14 days in osteogenic medium (DMEM additioned with 10% FBS, 1% HEPES, 1% pen strep, 15 mM ascorbic acid, 10 mM β - glycerophosphate, 10 nM dexamethasone, 10nM cholecalciferol). **A)** Alizarin red staining for the three different BMSCs populations; **B)** Expression of typical osteoblastic markers, normalized to MRC-5 expression (set to 1) and also quantified in RNA deriving directly from human bone samples (osteoblasts). *: p < 0.05, ***: p < 0.001 as compared to osteoblasts expression. All error bars represent standard deviations of at least three different experiments.

To establish a bone-like environment we differentiated BMSCs for 14 days in osteogenic medium, having previous surveys and literature data [22] established that it allowed to achieve adequate osteo-differentiation. However, considering the time-dependence of differentiation markers expression and the different effects reported on tumor cells co-cultured with variously differentiated bone cells [6], we determined the osteodifferentiation degree of BMSCs used in our experiments with reference to both terminally differentiated osteoblasts and MRC-5. We characterized BMSCs osteodifferentiation through Alkaline Phosphatase (ALP) assay, Alizarin red staining and calcium quantification. Moreover, expression of both early (RUNX-2) and late osteogenic markers (osteopontin and osteocalcin) [23], was determined. After 14 days in osteogenic medium, BMSCs from all the 3 different donors showed positive Alizarin red staining (Figure 3A), demonstrating the presence of calcium deposits. Their quantification demonstrated significantly higher calcium levels in BMSCs as compared to MRC-5 (p < 0.001, Additional file 2: Figure S1A). ALP activity, index of initial osteogenic commitment of cells, normalized to total protein content, was detectable in all the 3 patients at comparable levels as shown in Additional file 2: Figure S1B. Moreover, the expression of osteogenic markers osteopontin, osteocalcin and RUNX-2 was significantly up-regulated in osteo-differentiated BMSCs as compared to MRC-5. Osteopontin and osteocalcin expression was significantly lower than in primary osteoblasts (p < 0.05) whereas expression of RUNX-2 was comparable (Figure 3B).

In conclusion, our simple co-culture model allowed to obtain results otherwise not possible to achieve in *in vivo* experiments, whereby it would be impossible to sort, separate and analyze the cell populations involved in the metastatic process. In particular, we demonstrated for the first time that direct but not indirect co-culture between human tumor cells and primary bone-like cells induced a significant up-regulation of RANKL/OPG expression in bone metastatic cells, suggesting an important role of direct contact in the bone metastatic process. This is in line with the reported clinical expression, determined by immunohistochemistry, of RANKL in human bone metastases [24, 25]. Furthermore, although we do not yet have a mechanistic explanation, upregulation of RANKL/OPG ratio and BOKL clustering observed only in direct co-culture with osteo-differentiated BMSCs and not in culture with conditioned medium or in direct co-culture with fibroblasts, further support the hypothesis that heterotypic cell interactions between tumor cells and bone cells can have a key role in the establishment of bone metastases.

## Electronic supplementary material

Additional file 1: **Materials and methods.** Detailed information about cell isolation, differentiation and culture, gene expression and time lapse analyses. (DOCX 22 KB)

Additional file 2: Figure S1: Confirmation of osteo-differentiation of BMSCs. A) Quantification of calcium deposits for BMSCs from three different patients. ***: p < 0.001, compared to fibroblasts. B) Quantification of ALP activity, normalized to the total protein content. All error bars represent standard deviations of at least three different experiments. (PPT 188 KB)

## Abbreviations

BMSCs: Bone marrow stromal cells
BOKL: MDA-MB231-BO-KL
CM: Conditioned medium
GM: Growth medium.
