# Supplementary material for: Direct but not indirect co-culture with osteogenically differentiated human bone marrow stromal cells increases RANKL/OPG ratio in human breast cancer cells generating bone metastases
Source: Mol Cancer. 2014 Oct 21;13:238. doi: 10.1186/1476-4598-13-238 (PMC4213507; doi:10.1186/1476-4598-13-238)
Supplement: Supplementary file 1 — Additional file 1: Materials and methods. Detailed information about cell isolation, differentiation and culture, gene expression and time lapse analyses. (DOCX 22 KB) [file 12943_2014_1438_MOESM1_ESM.docx]

**Additional file 1: Supplementary materials and methods.**

**BMSCs isolation, differentiation and characterization**

BMSCs from 3 patients undergoing hip surgery after informed consent were isolated by centrifugation at 510g for 10 minutes and incubated in complete medium (α-MEM, 10% FBS, 100 U/ml Penicillin, 100 ug/ml Streptomycin, 1% HEPES, b-FGF 5ng/ml). Non-adherent cells were removed after 6 days and medium was changed twice a week until confluence. To induce osteo-differentiation, BMSCs were seeded at 3000 cells/cm^2^ in complete medium for 3 days. Osteogenic medium (OM) was successively added for 14 days: DMEM with 10% FBS, 1% HEPES, 1% sodium pyruvate, 100 U/ml Penicillin, 100 ug/ml Streptomycin, 2mM L-Glutamine, 0.15mM Ascorbic Acid-2P, 10mM β-glycerophosphate, 10nM Cholecalciferol, 10nM Dexhametasone, as already reported[15]. Medium was changed every 3-4 days. BMSCs osteodifferentiation level was characterized through Alkaline Phosphatase (ALP) assay, Alizarin red staining and calcium quantification by means of a commercial kit (RANDOX). Osteogenic marker expression (osteopontin, osteocalcin and RUNX-2) was determined with Real time PCR compared to a human fetal lung fibroblasts cell line (MRC-5) and to primary human osteoblasts, also derived from hip surgery patients.

**Tumor and fibroblast-like cell lines culture**

MDA-MB-231-BO, a bone metastatic breast cancer cell line derived from MDA-MB-231has been developed[[14](#_ENREF_14)] and kindly provided by Professor T. Yoneda (University of texas Health Science Cancer at San Antonio, USA). MDA-MB-231-BO cells have been transfected with Katushka plasmid (Evrogen), encoding for a red fluorescent protein and with PGL3-Luc plasmid, encoding for Firefly Luciferase enzyme. Clones expressing high levels of Katushka have been isolated by FACS and one of these (BOKL) was used in subsequent experiments. BOKL were cultured in growth medium (GM): DMEM with 10% FBS, 1% Penicillin/Streptomycin and 2mM L-Glutamine. MRC-5 fibroblasts were grown in EMEM with 10% FBS, 1% Penicillin/Streptomycin and 2mM L-Glutamine.

**Co-culture between BMSCs or MRC-5 and BOKL**

To study the effect of direct contact of tumor cells (BOKL) on bone-like cells (osteo-differentiated BMSCs) or MRC-5, five different experimental conditions were compared:

1. **BOKL in direct co-culture with osteo-differentiated BMSCs (BOKL co-culture BMSCs):** After BMSCs osteo-differentiation for 14 days, BOKL (5000 cells/cm^2^) were seeded on the BMSCs monolayer. BOKL adhered to BMSCs and both cell types were cultured in GM for 3 days.
2. **BOKL in conditioned medium from osteo-differentiated BMSCs (BOKL CM BMSCs):** After BMSCs osteo-differentiation for 14 days, osteogenic medium was removed; GM was then added for 24h to obtain conditioned medium (CM) from osteo-differentiated BMSCs. BOKL were seeded in a multiwell at 5000 cells/cm^2^ and cultured in 100% CM for 3 days.
3. **BOKL in co-culture with MRC-5 (BOKL co-culture MRC-5):** MRC-5 were kept in culture until confluence, then BOKL (5000 cells/cm^2^) were seeded on the MRC-5 monolayer and GM was added for 3 days.
4. **BOKL in conditioned medium from MRC-5 (BOKL CM MRC-5):** After MRC-5 culture until confluence, MRC-5 culture medium was removed and fresh medium was added for 24h to obtain conditioned medium (CM) from MRC-5. BOKL were seeded in a multiwell at 5000 cells/cm^2^ and cultured in 100% CM for 3 days.
5. **BOKL control:** As a control condition, BOKL were seeded at 5000 cells/cm^2^ in a multiwell and cultured for 3 days in GM.

**Gene expression analysis**:

After direct co-culture between BOKL and osteo-differentiated BMSCs or MRC-5, cells were enzymatically digested for 15 minutes with collagenase I (15 mg/ml) and for 15 minutes with trypsin-EDTA 0.05% at 37°C, under gentle agitation. Cells were then re-suspended in FACS buffer and subjected to FACS sorting, to obtain two separated populations.

Total RNA was isolated from individual cell populations using SV total RNA (Promega) and reverse transcribed (High-capacity cDNA archive kit - Applied Biosystem). Gene expression was assessed in relative quantitative RT-PCR with taqman^®^ probes (Applied Biosystem) for the target genes RANK (Hs00187192_m1), RANKL (Hs00243522_m1), OPG (Hs00900358_m1), CDH11 (Hs00901475_m1), RUNX2 (HS00231692_m1). As housekeeping gene human cyclofillin (4326316E-1101014) was used.

To exclude that modifications in gene expression were due to the above reported separation procedure and not to co-culture, data were normalized to appropriate controls. Gene expression levels of cells after co-culture were normalized respective to cells grown in GM and then subjected to the same enzymatic digestion and sorting procedure (control 1). For cells grown in CM, gene expression was instead normalized to BOKL control (control 2).

**Cell proliferation**

BOKL were seeded 5000/cm^2^ and grown for 3 days in the four different experimental conditions. After detachment with trypsin-EDTA 0.05%, cells were counted by an automated image-based Cell Counter, equipped with fluorescence filters (TALI^®^, LifeTechnologies), which allowed us to quantify separately red fluorescent BOKL and non-fluorescent BMSCs or fibroblasts.

**Time lapse analysis**

To monitor BOKL migration in the different experimental conditions, time lapse analysis was performed over 3 days, capturing an image every 15 minutes. A total of 10404 images were analyzed, specifically 9 fields for each of the four experimental conditions, 289 images for each field. ImageJ software-Manual Tracking tool was applied to measure the displacement of each cell between two consecutive images. To establish the existence of a preferential direction in cell migration, index of directionality P=A1/A2[25] was calculated, whereby A2 is the sum of distances traveled by each cell through all the 289 images whereas A1 is the distance, calculated for each cell, between the initial position (first image) and the final one (last image). P values near 0 indicate a random movement, whereas higher P values indicate a preferential migration.

To quantify cell clusterization at the end of the co-culture, the image at 72h time point was analyzed, counting cells. Clusters were defined as groups of at least 4 cells in direct contact, a definition already adopted for micro-metastases[24]. Cluster number was normalized to total cell number, to exclude the possible effect of cell proliferation.

**Statistical analysis**

All statistical analyses were performed with Prism5. ANOVA followed by Bonferroni post-hoc test was used to detect significant differences between groups. Level of significance was set at p<0.05.
