# Supplementary figures and images for: Direct but not indirect co-culture with osteogenically differentiated human bone marrow stromal cells increases RANKL/OPG ratio in human breast cancer cells generating bone metastases
Source: Mol Cancer. 2014 Oct 21;13:238. doi: 10.1186/1476-4598-13-238 (PMC4213507; doi:10.1186/1476-4598-13-238)

## Slide 1
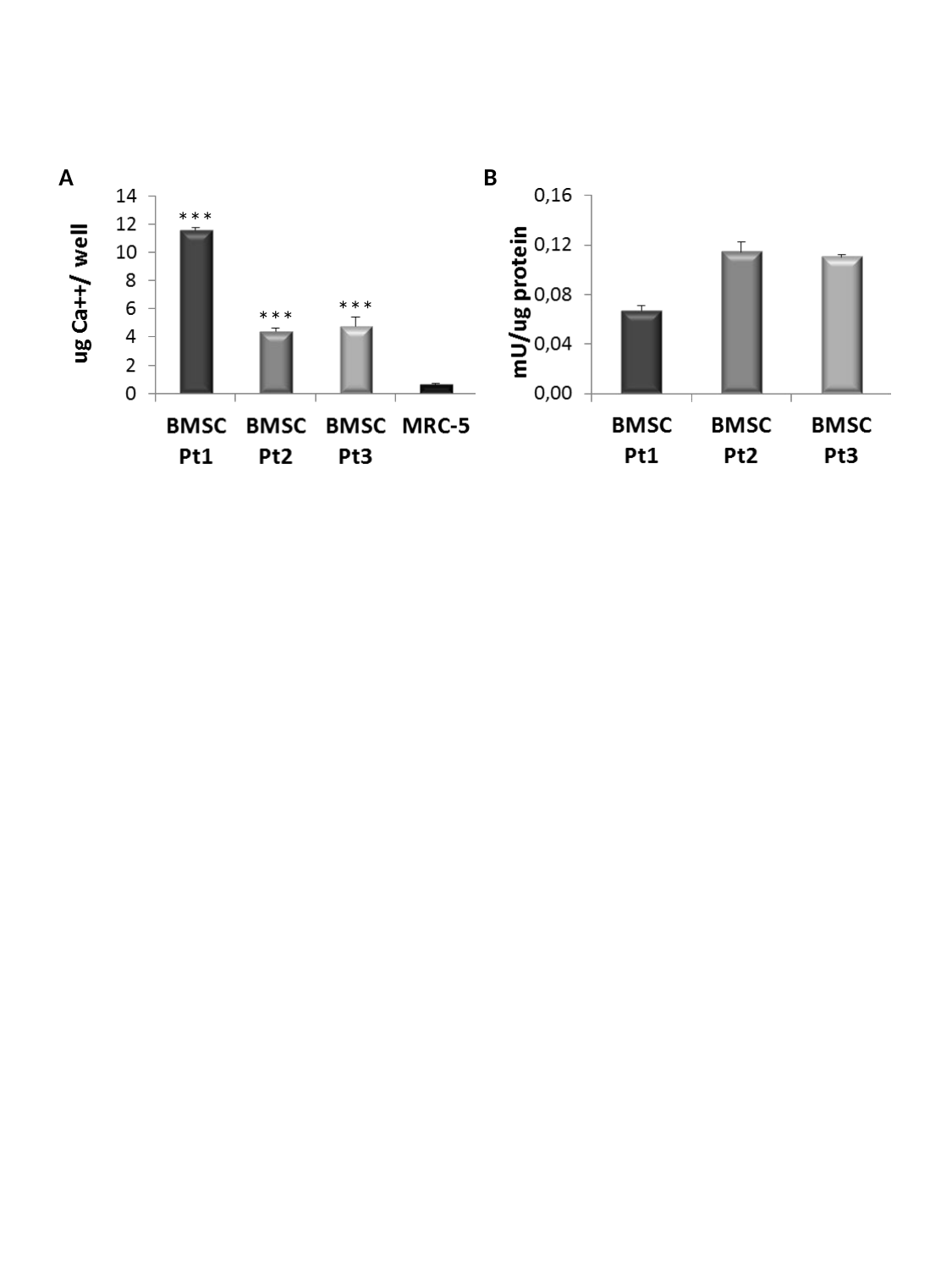

A
B
***
***
***

Supplement: Supplementary file 2 — Additional file 2: Figure S1: Confirmation of osteo-differentiation of BMSCs. A) Quantification of calcium deposits for BMSCs from three different patients. ***: p < 0.001, compared to fibroblasts. B) Quantification of ALP activity, normalized to the total protein content. All error bars represent standard deviations of at least three different experiments. (PPT 188 KB) [file 12943_2014_1438_MOESM2_ESM.ppt]
